# Gastrointestinal basidiobolomycosis – A rare fungal infection: Challenging to diagnose yet treatable – Case report and literature review

**DOI:** 10.1016/j.idcr.2023.e01802

**Published:** 2023-05-21

**Authors:** Saeed Mirmoosavi, Mohammadreza Salehi, Reza Fatahi, Amanuel Godana Arero, Hasti Kamali Sarvestani, Farid Azmoudeh-Ardalan, Faeze Salahshour, Masoomeh Safaei, Sara Ghaderkhani, Foroogh Alborzi Avanaki

**Affiliations:** a Endocrinology and Metabolism Research Center (EMRC), Vali-Asr Hospital, Tehran University of Medical Sciences, Tehran, Islamic Republic of Iran; b Research Center for Antibiotic Stewardship & Antimicrobial Resistance, Department of Infectious Diseases, Tehran University of Medical Sciences, Tehran, Islamic Republic of Iran; c Cardiac Primary Prevention Research Center, Cardiovascular Diseases Research Institute, Tehran University of Medical Sciences, Tehran, Islamic Republic of Iran; d Department of Medical Parasitology and Mycology, School of Public Health, Tehran University of Medical Sciences, Tehran, Islamic Republic of Iran; e Imam Khomeini Hospital, Tehran University of Medical Sciences, Tehran, Islamic Republic of Iran; f Advance Thoracic Research Center, Tehran University of Medical Sciences, Tehran, Islamic Republic of Iran; g Department of Pathology, Cancer Institute, Imam Khomeini Hospital Complex, Tehran University of Medical Sciences, Tehran, Islamic Republic of Iran; h Department of Infectious Diseases, Imam Khomeini Hospital Complex, Tehran University of Medical Sciences, Tehran, Islamic Republic of Iran

**Keywords:** Basidiobolomycosis, Crohn’s disease, Gastrointestinal obstruction, Inflammatory bowel disease, Gastrointestinal basidiobolomycosis, Fungal infection

## Abstract

Gastrointestinal Basidiobolomycosis is a rare manifestation of Basidiobolus ranarum infection. In this report, we present two cases of gastrointestinal Basidiobolomycosis. The first patient presented with obstructive symptoms, fever, and weight loss. The diagnosis of Basidiobolomycosis was not made until after surgery, when Liposomal amphotericin-B combined with itraconazole were administered, leading to the resolution of laboratory markers of inflammation and patient's symptoms. The second case involves a young woman who presented with hematochezia, perianal induration, and abdominal pain. The patient had previously been diagnosed with Crohn's disease and treated accordingly, but her symptoms did not improve. Due to the endemicity of tuberculosis in Iran, the patient was treated for TB but still showed no improvement. However, a perianal biopsy sample revealed the Splendore Hoeppli phenomenon and fungal elements in GMS staining, leading to the diagnosis of gastrointestinal Basidiobolomycosis. Treatment with itraconazole and co-trimoxazole led to a significant improvement in symptoms and laboratory indices after one week, including the resolution of perianal induration. The key takeaway from this report is the importance of considering rare infections in the differential diagnosis of gastrointestinal conditions such as IBD and GI obstruction.

## Introduction

There are more than 100,000 fungi worldwide, but only about 150 of them are pathogenic [1]. *Basidiobolus ranarum* is a filamentous fungus in the Entomophthorales group of the Zygomycota family that has been associated with emergent fungal infection characterized by chronic subcutaneous induration [2], [3]. It is predominantly found in tropical parts of South America, Africa, and Asia [4]. *B. ranarum* is a saprophyte found in soil and decaying vegetable [5]. Certain amphibians, fish, reptiles, and insectivorous bats have this organism as a commensal [2]. In humans, *Basidiobolus* sp. infection is destructive and lethal [6]. The mode of transmission to humans is yet unclear. For the subcutaneous form, skin penetration following a minor skin injury, scratches, and insect bites are a few potential entry points for infection [7]. Although visceral involvement with *Basidiobolus sp.* is rare, ingestion of contaminated soil, animal feces, or food is a possible route of infection [7], [8].

*B. ranarum* was found in frogs in 1886 primarily and it is an environmental saprophyte found in soil and decaying vegetables [2], [9]. Infection is acquired through eating contaminated food, penetration in skin caused by trauma or bite, and inhalation of spores or their introduction into the nasal cavities by soiled hands [10]. The Zygomycetes contain two fungal patterns: *Mucorales* and *Entomophthorales*, with totally dissimilar pathogenic capacities. *Mucorales* order take in the immunocompromised individuals, whereas *Entomophthorales* which contain *Basidiobolus genera* are the reason for infection in immune-competent patients [2], [7], [8]. This fungal infection is very rare in immunocompromised hosts like diabetics and renal transplant patients [2], [10].

Gastrointestinal basidiobolomycosis (GIB) is an exceedingly uncommon presentation of *basidiobolus* sp. infection. The first presumed case was reported in Nigeria in 1964 [2]. It typically involves the liver, colon, and small intestine and in some cases, it contaminates the biliary tract and pancreas [5], [10], [11]. The majority of cases that have been reported have been from Middle East countries (Iran, Saudi Arabia, and Kuwait) [5], [8], [12]. Initial suspicion for GIB because of its paucity of cases is not possible and no certain and specific clinical presentation is found and all earlier cases have been managed without a primary diagnosis of GIB [13], [14], [15]. The clinical and radiological findings of GIB usually mimic neoplastic or inflammatory bowel conditions [9], [12], [15], [16]. The diagnosis of GIB is based on histological findings (granulomatous reaction, dense infiltrate of eosinophils, and fungal structures) [17], [18]. To improve our knowledge of the clinical manifestations, and therapeutic options for basidiobolomycosis, here we report the clinicopathologic characteristics of two GIB patients.

## Case presentation

### Case 1

A 46-year-old man presented to our emergency department with colicky lower abdominal pain for 4 months. The pain was hypogastric, colicky in nature, and increasing in intensity. It was incompletely relieved by using analgesics. He had mucoid diarrhea for 2 months. He had nausea and vomiting containing food particles but did not report hematemesis. The patient also complained of recurrent attacks of fever, fatigue, and weight loss of more than 20 kg over the previous 4 months. He was an addicted to inhaled opium since ten years ago, but his history was not remarkable for any tumor or malignancy.

On physical examination, the patient was mildly dehydrated. Blood pressure was 110/75 mmHg; heart rate, 84 beats per minute; respiratory rate, 19 per minute; temperature 36.7 ◦C; the patient had tenderness in palpation of the abdomen at RLQ. Multiple masses were palpable in the right side of the abdomen and they measured about 30 * 30 mm in size and the percussion was dull. The laboratory results are shown in Table 1.

**Table 1:** Laboratory test results of both patients.

Test description	Observed		Value	
	During hospitalization		After discharge	
	Case 1	Case 2	Case 1	Case 2
WBC	12.3 * 10^3^ µL	20.19 * 10^3^ µL	5.8 * 10^3^ µL	4 * 10^3^ µL
Neutrophil	75.5 %	88 %	46.7 %	52 %
lymphocytes	21.4 %	6.7 %	38.5 %	34 %
Eosinophil	2.5 %	2.7 %	12 %	11 %
Monocyte	0.6 %	1.7 %	2.8 %	3 %
Hgb	10.7 g/dl	6.6 g/dl	11.2 g/dl	10.1 g/dl
Hct	33.4 %	21 %	38.2 %	33 %
MCV	83.5 fL	61 fL	86.7 fL	71 fL
PLT	919 * 10^3^ µL	506 * 10^3^ µL	287 * 10^3^ µL	332 * 10^3^ µL
ESR	40 mm/h	30 mm/h	18 mm/h	27 mm/h

Note: White blood cells (WBC), Hemoglobin (Hgb), Hematocrit (Hgb), mean corpuscular volume (MCV), Platelet (PLT), erythrocyte sedimentation rate (ESR), Microlitre (µL), Femtolitre (fL), grams per deciliter(g/dl), Millimeters per hour(mm/h).

**Fig. 1:**
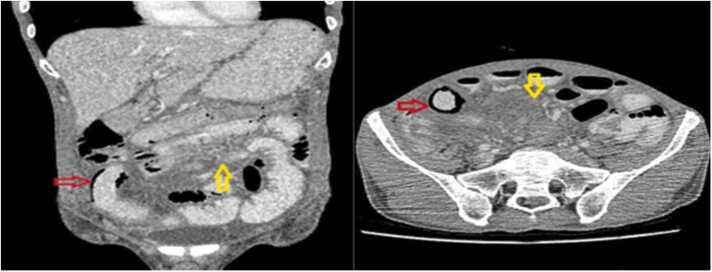
Axial and coronal contrast-enhanced abdominopelvic CT scan depicts increased density of ileal mesentery, representing an infiltrative process (yellow arrow). An ileal loop shows pneumatosis intestinalis in RLQ suggestive of transmural bowel necrosis (red arrow).

He had a duodenal ulcer in endoscopy and two times normal colonoscopy. Ultrasonography detected free liquid collection in the pelvic cavity. A subsequent CT scan revealed Soft tissue density in the distal ileum, free liquid in the peritoneum, and parietal pneumatosis (5 cm in length) was detected in one of the ileal loops resembling necrosis. CT findings were similar to desmoid tumors or carcinoids. No major pathology was detected in other viscera of the abdomen (Fig. 1).

Due to presence of obstructive symptoms, and suspected abdominal infection, the patient underwent antibiotic therapy with cefepime (2 g/IV/TDS) and segmental resection surgery of the ileum. The resected segment was sent for histopathologic evaluation. Three intestinal wall perforations were seen. The intestinal wall was thin and the external surface was covered by whitish-brownish fibrin.

**Fig. 2:**
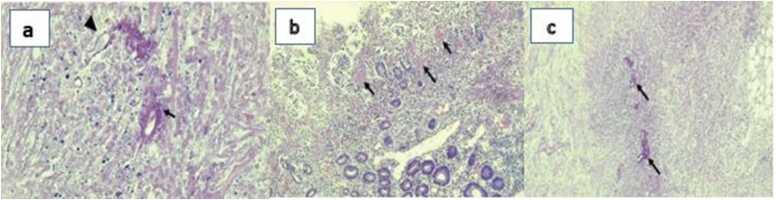
**a**: The organisms are broad and aseptate (arrowhead), and are surrounded by an eosinophilic material (Splendore-Hoeppli reaction) (arrow), (H&E, 400 ×). **b**: Mucosal necrosis and capillary micro thrombi (arrow) in favor of ischemic pattern of injury are identified. (H&E, 100 ×). **c**: Necrosis and inflammatory response and fungal organisms (arrow) are seen in the mesenteric mass. (H&E) element (red arrow). H&E stain, 100 ×. **C**. a multinucleated giant cell with an ingested fungal element. H&E 1000 ×. The inset highlights the micro-organism by special staining. Gomori Methenamine Silver (GMS) stain, 1000 ×. The yellow arrows point to the micro-organism. **D**. Splendore Hoeppli phenomenon. H&E stain, 1000 ×.

The histopathology report showed multiple organisms associated with Splendore-Hoeppli reaction, necrosis, inflammatory response, foci of increased eosinophils, and granulomatous inflammation. These findings were consistent with basidiobolomycosis. An extension of organisms into the blood vessel walls was identified (Fig. 2a). Sub mucosal hyalinization of thrombotic blood vessels and some irregular crypts in favor of ischemic ileitis were evident (Fig. 2b). Two surgical end margins showed neutrophilic exudate and supportive inflammation in the serosa. Four lymph nodes were detected. One of them showed extensive necrosis and scattered basidiobolomycosis associated with granulomatous inflammation (Fig. 2c).

Subsequently Liposomal amphotericin-B 200 mg/day in combined with itraconazole 600 mg/Orally/daily were started. CRP and ESR level deceased and patient's signs and symptoms improved. Unfortunately, the patient expired a month and a half after recovery due to opium overdose and subsequent apnea.

### Case 2

**Fig. 3:**
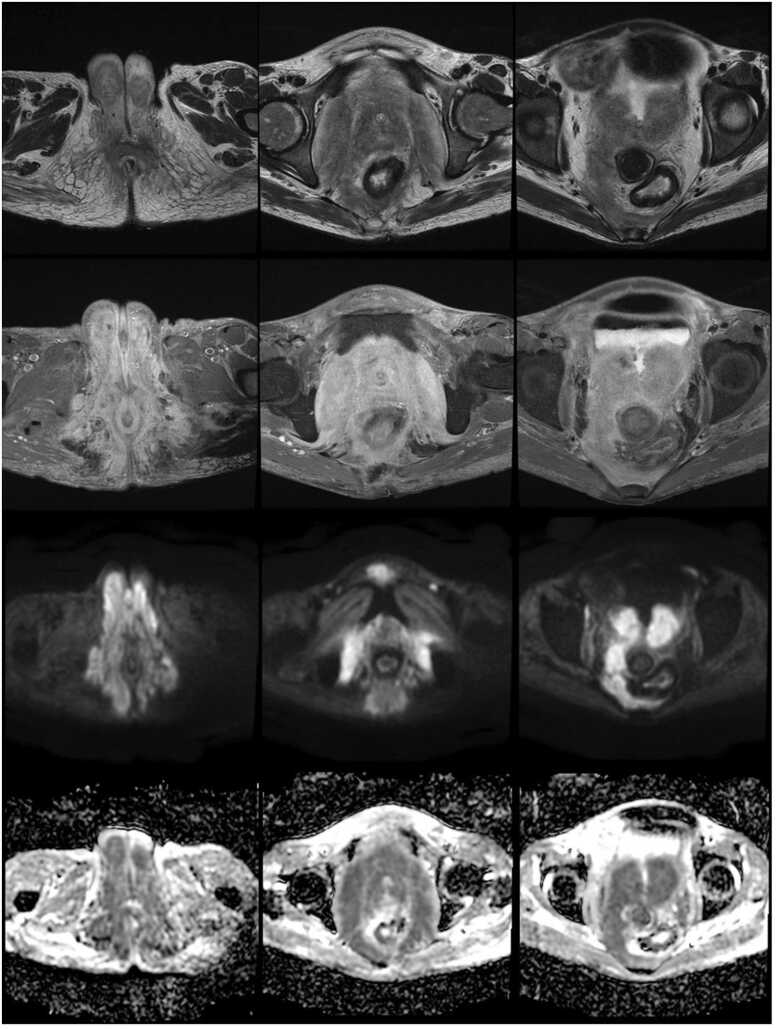
An infiltrative soft tissue is seen in the pelvic and perineum. It infiltrates the perineal skin and both labia major, and bilateral internal obturator muscles leading to the enlargement of these structures. presacral fat, and mesorectal fascia in the right side and the bladder neck were also involved. The lesion had an iso signal on T1 weighted, and a mildly high signal on T2 weighted images, showing mild enhancement and restriction of diffusion on DWI sequences. (from top to bottom first row: T2 weighted, second row: T1 weighted with contrast,third row: DWI and the fourth row:ADC).

A 35 years-old woman from Kerman province has come to our clinic with abdominal pain mainly in the hypogastric area, constipation, and hematochezia. colonoscopy showed multiple erosions and ulcers in the sigmoid and descending colon with skip area. Based on colonoscopy and histopathology she had been diagnosed with Crohn's disease and mesalamine was prescribed for her. After a while due to incomplete symptom response. Her physician initiated Adalimumab. After initiation of Adalimumab, she noticed abdominal distension. At this time, she was referred to a tertiary center for more evaluation. With the impression of refractory Crohn's disease infliximab (Remicade) was started. Starting remicade was followed by increasing abdominal distension and perianal induration. MRI revealed sinusitis and extensive inflammation and swelling in pelvic floor muscles leading to anal canal narrowing. It also showed pan colitis and terminal ileitis with an active pattern in the rectosigmoid (Fig. 3).

The patient underwent a surgical biopsy of perianal tissue. microscopic examination showed poorly formed granuloma with central necrosis and occasional PMN aggregates and prominent infiltration of eosinophils. Ziehl-Neelsen and PAS staining were negative for acid-fast bacilli and fungal elements respectively. According to the finding of granulomatosis reaction and endemicity of TB in IRAN and the fact that symptoms worsened after anti-TNF therapy, anti-TB treatment was started at that center. She was referred to our hospital at this stage which she developed acute drug-induced hepatitis.

Despite anti-TB medications, there was still leukocytosis and elevated ESR and CRP. Our DDxs at this time were: pelvic Wegner disease, Histiocytosis X, lymphoma, Crohn's disease with cutaneous involvement and rare causes of gastrointestinal granulomatosis such as syphilis, Bartonella henselae, Basidiobolomycosis, and salmonellosis. Although we had negative results for fungal infection we started empirical amphotericin. No improvement was reached after two weeks. cystoscopy showed severe inflammation and erythema with severe PMN infiltration. Bone marrow biopsy was negative for infiltrative disease such as lymphoma. Laparoscopic exploration revealed distended bowel loops but no obvious mass. The laboratory results are in Table 1.

**Fig. 4:**
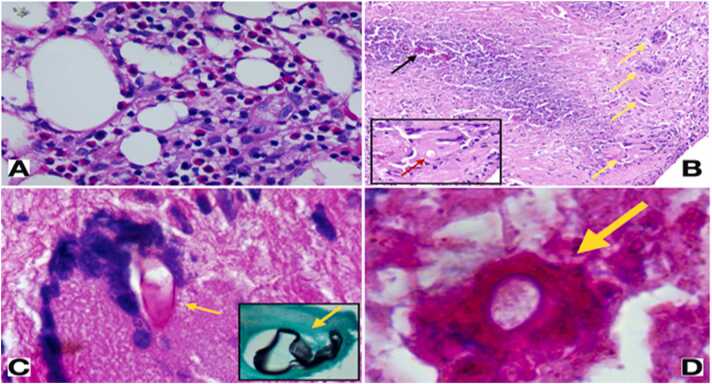
Histopathology of perineal biopsies taken under CT guide. **A**. Panniculitis with marked eosinophilic infiltration. H&E stain, 100 ×. **B**. Granulomatous reaction with necrosis. The yellow arrows point to multinucleated giant cell. The black arrow depicts the necrotic areas and the Splendore Hoeppli phenomenon H&E stain, 40 ×. The inset depicts a high-power view of the giant cell bearing a fungal.

We reviewed all of the patient’s histopathology samples to come up with a diagnosis with another expert pathologist. The pathologist diagnosed basidiobolomycosis by viewing the Splendore Hoeppli phenomenon and fungal element in GMS staining in samples of perianal biopsy (Fig. 4).

**Fig. 5:**
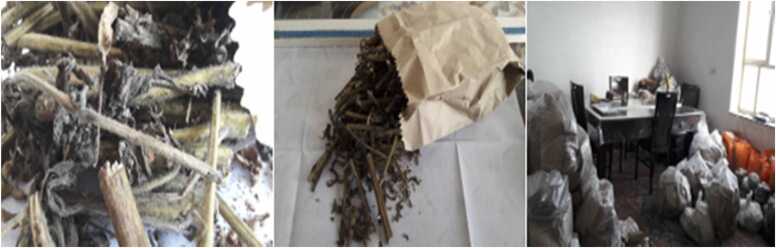
Photos from patient’s home showing sesame cultivation.

**Fig. 6:**
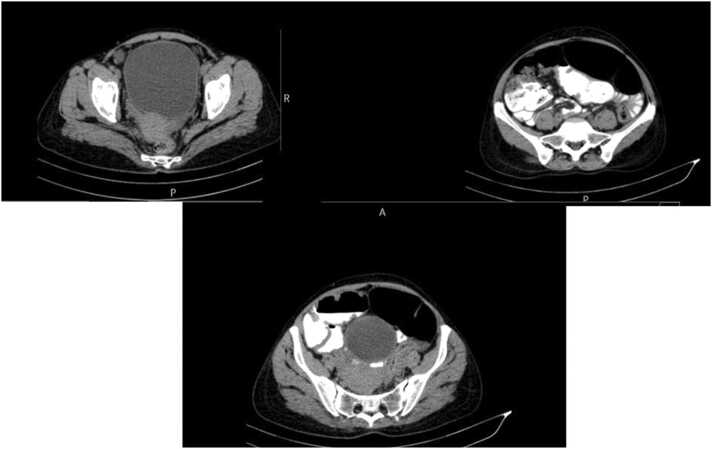
Abdominopelvic CT scan of the second patient after treatment shows resolution of inflammation and obstruction.

Itraconazole and co-trimoxazole were started after consultation with infectious disease specialists who had similar cases. One week later the patient’s symptoms and laboratory indices (ESR, CRP, anemia) improved significantly (Table 1). Perineal induration was also resolved. The patient was discharged three weeks later. Itraconazole and co-trimoxazole were continued for 9 months. We were curious about the source of the infection and after asking for possible exposures we found out that the patient had been culturing sesame in her house for her Ph.D. thesis (Fig. 5). The only problem at the time of discharge was abdominal distention which had not been fully resolved. Three and twelve-month later at the follow-up CT scan there were no obstructions and the distension was attributed to the dysmotility of the sigmoid part of the colon which is a sequela of sigmoid involvement by basidiobolomycosis (Fig. 6). Now one year later after discharging the patient at the follow-up visits, she is in good condition.

## Discussion

In this article, we have presented two cases of gastrointestinal basidiobolomycosis with two different presentations during the last two years in our hospital. Crohn's disease mimicking disorder and GI obstruction.

The primary presentation of both cases was not suggestive of fungal disease. The first case underwent surgical operation due to obstructive symptoms with a primary diagnosis of malignancy in both imaging and surgical gross appearance with multiple abscess formation and severe adhesions and inflammation. The diagnosis of the second case had been delayed for about 10 months. Primary presentation, the endoscopic appearance of ulcers, and pathologic findings were not in favor of the disease. None of the typical pathologic findings of this disease have been found in primary specimens. She was misdiagnosed with Crohn's disease and after administration of anti-TNF, the patient developed severe perianal induration and panniculitis where finally the typical pathologic findings have been documented in this specimen after multiple consults with pathologists. The absence of typical findings in colon specimens highlights the fact that in endemic areas such as some parts of Iran high clinical suspicion should be considered.

Basidiobolomycosis is caused by basidiobolus ranarum, a rare known fungal organism [19]. It is prevalent all over the world, however, the majority of cases have been documented in tropical and subtropical regions, with most cases reported from Saudi Arabia [19] and Iran [11], [13], [15].

Less than 80 case reports are published in total [4]. Most cases are reported from Middle East countries (Iran, Saudi Arabia, and Kuwait) [2], [12]. Immunocompetent individuals are at risk for this organism’s infection (2). Basidiobolomycosis in immunocompetent hosts has been reported in many countries: 23 reports from Saudi Arabia and the US, 17 from Iran, 2 from Kuwait, 6 from Iraq, 2 from Nigeria, 4 from Brazil, and 1 from the Netherlands [4], [12]. GIB does not show a predilection for any particular age group and has been reported in patients aged 1.5 years to 80 years [4]. Now available data recommends that males may be more susceptible than females; only 6 of the 80 reported cases were in females [3].

As a result, there is a reasonable suspicion that the warm and arid climate typical in these countries may be a contributing factor, though the precise mechanism is unclear [5], [10], [11]. Basidiobolomycosis typically causes a chronic subcutaneous infection that mostly affects the trunk and extremities [2]; however, in recent years there have been reports of visceral involvement, making this fungus infection potentially fatal [2], [8]. In the subcutaneous form of the infection, skin penetration following a minor skin injury, scratches, and insect bites are a few potential entry points for infection [7], [20]. Interestingly our patient developed skin induration after administration of anti-TNF. Our second presented case had been culturing sesame in her house for her Ph.D. thesis and raised the possibility of infecting the skin with polluted hands and ingesting the fungus. Ingestion of contaminated food with soil or animal faces is a potential route of infection for invasive visceral involvement, such as GIB (8, 10).

Gastrointestinal basidiobolomycosis is a very rare presentation of basidiobolus infection [8]. It typically affects the liver, colon, and small intestine, but it can also affect the biliary tract and pancreas [5], [7], [9], [10], [11]. According to a recent review conducted to examine the epidemiology, clinical manifestations, histopathology, management, and prognosis of GIB, the colons and rectum (82 %), small intestines (36 %), and liver and gallbladder (30 %) were the most common sites of involvement [4]. Two of our reported cases, wherein gastrointestinal involvement involved the colon and small intestines, are consistent with the aforementioned findings.

Gastrointestinal basidiobolomycosis presents with nonspecific manifestations [12]. The most common GIB symptoms include abdominal pain, constipation, abdominal distention, palpable abdominal mass, fever, and diarrhea [4]. The nonspecific characteristics of the symptoms may delay diagnosis and increase the patient's risk of morbidity. According to the above-mentioned review article, intraabdominal malignancy was the initial provisional diagnosis in most (43 %) patients prior to confirmation of GIB, followed by inflammatory bowel disease (16 %) and diverticulitis (11 %) [4]. Our findings in terms of clinical symptoms and preliminary diagnosis were congruent with the findings of these recent review articles. GIB laboratory findings may include an elevated white blood cell count, peripheral blood eosinophilia, and serum antibodies to *B. ranarum* [4]. Diagnosis of GIB is usually by fungal staining of biopsy tissue [18]. The presence of granulomatous inflammation, tissue eosinophilia, and the Splendore-Hoeppli phenomenon is GIB histopathological characteristics [4], [17], [18]. Imaging modalities, particularly computed tomography and endoscopy, may reveal the abdominal mass and infection spread throughout the body [4], [19]. Our reported case's histopathological, laboratory and imaging findings were consistent with previously reported cases [4], [19].

Definitive diagnosis requires a culture of the organism but, unfortunately, a fungal culture is positive in only 15–25 % of cases [15], [16]. Polymerase chain reaction can be helpful when fungal culture is negative. Molecular diagnostic tests are more accurate because DNA can be isolated even from formalin-fixed paraffin-embedded tissue [4], [12].

The risk for basidiobolomycosis may be higher in individuals with uncontrolled diabetes mellitus (particularly with ketoacidosis), prolonged neutropenia, prolonged corticosteroid use, hematological malignancy, organ transplant, iron overload, an acquired immunodeficiency syndrome (AIDS), injection drug use, trauma/burns, and malnutrition [2], [4], [10].

Since GIB is a life-threatening condition that can be fatal, treatment options must be carefully considered [2]. Unfortunately, there is little data on a specific treatment option for GIB. However, based on existing data, it appears that extended antifungal medication alone or in combination with surgical resection of masses and debridement of the affected tissue can be effective [4], [21], [22].

There are two approaches to the treatment of GIB. Immediate initiation of liposomal amphotericin-B with the mechanism of binding to ergosterol in the fungal cell membrane, which leads to the formation of pores, ion leakage, and ultimately fungal cell death that we prescribed for our patient is preferred clinically [4], [6], [7]. Triazoles with the mechanism of blocking the cytochrome P450-dependent enzyme C-14 alpha-demethylase, which is needed to convert lanosterol to ergosterol, like fluconazole, itraconazole, voriconazole, and caspofungin are Antifungal agents that have not shown efficacy against *Mucor* in clinical, and in vitro studies [21]. Posaconazole or isavuconazole with the mechanism of disrupting the biosynthesis of ergosterol, which is a key component of the fungal cell membrane is used as maintenance therapy for patients who have responded to amphotericin-B [23].

In our reported case, one of the patients received only antifungal medication whereas the other patient received both surgical resection and antifungal medication. Both of our patients had a favorable outcome. Uncertainty exists about the precise role that surgical excision plays in the treatment of GIB patients [4]. For this reason, while it must be done case-by-case, we recommend considering medical therapy before surgical interventions. We had to operate on the first case due to obstructive symptoms and anti-fungal initiated after the establishment of the diagnosis by the pathologist. The second case responded dramatically to itraconazole. After one and a half years she was in good condition except for bowel distention which the radiologist reported her to follow up CT as nonfunctional sigmoid without any sign of inflammation. In her follow up she also had elevated ferritin in spite of normal ESR and CRP. She is considered for surgery and resection of the involved segment of the colon and it seems that surgery is one of the cornerstones of treatment.

There is limited information available on the type of antifungal agents and their duration [23]. Itraconazole continues to be the most successful treatment for GIB, accompanied by surgical excision of the mass [4], [19]. Among the azoles, fluconazole and ketoconazole have been demonstrated to be beneficial in select cases of subcutaneous forms [24]. Additionally, there are few cases of posaconazole successfully treating GIB [23], [25]. The recommended dosage of itraconazole is 100 mg twice daily for a period of four to twelve months [26]. In our case, itraconazole was given to one patient along with surgical resection of the mass, while liposomal amphotericin B was given to the other patient. Findings from our mentioned report underline the necessity of liposomal amphotericin B for the treatment of GIB where Itraconazole is contraindicated or unavailable, based on a favorable outcome following both types of antifungal drugs. A combined regimen of itraconazole and liposomal amphotericin B might possibly be helpful, although we need data to support this claim.

In patients who do not respond to, or cannot tolerate, amphotericin B, Both drugs can also be used as second-line therapy [18]. if there is a clinical suspicion of GIB, empirical therapy with antifungal agents should be prescribed [1]. An additional medical treatment modality is Oral saturated solution of potassium iodide (SSKI), particularly before the availability of azoles; which is now replaced by itraconazole but it may still be prescribed in developing countries due to low cost. Side-effects such as metallic taste, emesis, and salivary gland enlargement may limit its use [27]. Co-trimoxazole also has been reported to be effective in some cases of basidiobolomycosis probably due to its antifungal activities; it could be used as an empiric therapy and in cases which do not tolerate Itraconazole [28], [29].

Patients may develop uni or multi colonic or liver or porta hepatic masses due to the progressive nature of basidiobolomycosis which can get disseminated. Therefore, early diagnosis and surgical intervention to avoid complications are necessary and long-term follow with the patients is required.

## Conclusion

GIB is more frequent in tropical and subtropical regions of the world and is characterized by nonspecific symptoms and lab findings that might mimic malignancy or inflammatory bowel disease. Physicians, especially those practicing in areas where the disease is epidemiologically prevalent, can make prompt diagnoses and provide effective treatments by being aware of the disease's clinical presentation, radiographic, endoscopic findings, and typical histopathologic findings. Diagnosis of this disease needs high suspicion from the physician. It is necessary to conduct further studies on environmental reservoirs, disease risk factors, and predictors of unfavorable outcomes.

## CRediT authorship contribution statement

**Saeed Mirmoosavi:** Writing – review & editing. **Mohammadreza Salehi:** Writing – review & editing. **Reza Fatahi:** Writing case presentation of case 1 and introduction. **Amanuel Godana Arero:** Writing the introduction and discussion . **Hasti Kamali Sarvestani:** Writing – review & editing. **Farid Azmoudeh-Ardalan:** Writing – review & editing. **Sara Ghaderkhani:** Writing – review & editing. **Foroogh Alborzi Avanaki:** Writing – review & editing.

## Ethical approval

This study was in concordance with Helsinki declaration of ethical principles for medical research involving human subjects.

## Consent

Written informed consent was obtained from the patient for publication of this case report and accompanying images. A copy of the written consent is available for review by the Editor-in-Chief of this journal on request. Unfortunately, the first patient of this study died prior to writing this case report and we were unable to obtain a written consent from him.

## Funding

This study didn’t have any funding source.

## Conflict of interest

The authors declare no conflicts of interest.

## Data Availability

Data of patients presented in this report will be available on a reasonable request.
